# Association between Child Abuse and Poor Oral Habits in Mongolian Adolescents

**DOI:** 10.3390/ijerph191710667

**Published:** 2022-08-26

**Authors:** Aina Okawara, Yusuke Matsuyama, Miyu Yoshizawa Araki, Yuko Unnai Yasuda, Takuya Ogawa, Tsasan Tumurkhuu, Ganjargal Ganburged, Amarsaikhan Bazar, Takeo Fujiwara, Keiji Moriyama

**Affiliations:** 1 Department of Maxillofacial Orthognathics, Graduate School of Medical and Dental Sciences, Tokyo Medical and Dental University, 1-5-45 Yushima, Bunkyo-ku, Tokyo 113-8519, Japan; 2 Department of Global Health Promotion, Graduate School of Medical and Dental Sciences, Tokyo Medical and Dental University, 1-5-45 Yushima, Bunkyo-ku, Tokyo 113-8519, Japan; 3 Department of Orthodontics, School of Dentistry, Mongolian National University of Medical Sciences, Ulaanbaatar 14210, Mongolia; 4 Department of Prosthodontics, School of Dentistry, Mongolian National University of Medical Sciences, Ulaanbaatar 14210, Mongolia

**Keywords:** child abuse, poor oral habits, bruxism

## Abstract

(1) This study aimed to investigate the association between child abuse and oral habits in adolescents in Mongolia. (2) A cross-sectional survey was conducted with children and their caregivers in Ulaanbaatar, Mongolia. Parents of 770 children enrolled in two public schools in Ulaanbaatar, Mongolia, completed questionnaires regarding the physical and psychological abuse that their children were subjected to and the presence of poor oral habits (biting nails/lips/pens and bruxism). Multivariable Poisson regression models were fitted with adjustment for age, gender, age of the mother, parental education, family income level, birth order, and living status with grandparents. (3) Biting nails/lips/pens and bruxism were reported by 39.0% and 17.5% of the respondents, respectively. Biting nails/lips/pens was significantly associated with physical abuse but was not significantly associated with psychological abuse (prevalence ratio, PR [95% confidence interval, CI]: 1.44 [1.07–1.95] and 1.34 [0.98–1.83], respectively). However, bruxism was not associated with physical or psychological abuse (PR [95% CI]: 1.16 [0.77–1.77] and 1.04 [0.68–1.61], respectively). (4) Child abuse was associated with biting habits among Mongolian adolescents.

## 1. Introduction

Poor oral habits, such as biting the lips and nails, sucking fingers, breathing through the mouth, and abnormal swallowing, can increase oral problems. For example, the habits of protruding the tongue and breathing through the mouth are associated with Angle Class II division 1 malocclusions [1]. There is an increased prevalence of the presence of E. coli and total Enterobacteriaceae in the saliva of participants with nail-biting habits; hence, their contamination risk might be high [2]. In adolescents, bruxism increases the sensitivity of the mandibular joint to palpation and masticatory muscle pain during chewing [3]. Therefore, there is a need to identify and reduce the risk factors for poor oral habits.

Further, psychosocial factors have been reported to be associated with poor oral habits. Exposure to stress [4,5,6] and mental disorders [7,8] contribute to poor oral habits in adolescence. A systematic review reported that having anxiety, worry, emotional tension, and schizophrenia was associated with wake-time teeth clenching [9]. A Dutch study found that stress and depressed mood were significantly associated with self-reported sleep and awake bruxism [5]. Furthermore, a study performed on rats reported that repeated exposure to screams, urine, and feces odors was associated with increased bruxism-like episodes, suggesting that chronic stress resulting from emotional stimuli may cause non-functional masticatory movements [10]. This indicates that psychosocial factors, including stress and anxiety, contribute to poor oral habits. However, the association between oral habits and the root causes of stress and anxiety in adolescents, including poor parenting, remains unclear.

Child abuse can have significant lifelong health consequences, including depression and anxiety [11]. It is estimated that worldwide, three out of every four children (approximately 300 million children) aged 2–4 years suffer from abuse by their parents or guardians on a daily basis [12]. In particular, psychological abuse is the strongest risk factor for depression [13]. Therefore, child abuse may increase the risk of poor oral habits, for which psychosocial factors are considered risk factors. It has been reported that children of abuse in Iraq demonstrate an increased prevalence of thumb sucking and nail biting [14]. The study used the Glasser criteria, which comprises 44 questions to assess child abuse, and a questionnaire to assess oral habits that was answered by the participating children. However, this study did not consider potential confounding factors and only assessed the association of oral habits with psychological abuse. In a case-control study conducted in Brazil involving 362 subjects, children with a history of abuse were twice as likely to have an anterior open bite. This was attributed to the establishment and maintenance of oral habits, including finger sucking, in family environments that create stress and anxiety in the child, and it has been suggested that the frequency of such habits may be higher in cases of child abuse [15]. However, these previous studies did not consider the type and the number of types of abuse. Several studies have demonstrated that different types [13] and the number of types [16,17,18,19,20] of abuse have different effects on health behaviors; therefore, there is a need to investigate the risk of oral habits according to the type and number of types of child abuse.

Mongolia became a full democracy in 1992. The social environment changed dramatically with the transition from the socialist system, which may have affected adolescents and their parents. The Multiple Indicator Cluster Survey conducted by the Mongolian National Statistical Committee and UNICEF in 2013 found that 46.9% of Mongolian children aged 1–14 years experienced psychological or physical disciplining [21]. In 2016, the Child Protection Law was enacted to prohibit the physical punishment of children in Mongolia. However, there was no decrease in the percentage of children experiencing physical and psychological disciplining in 2018. Thus, the association between child abuse and oral habits can be evaluated in Mongolian adolescents. The aim of this study was to investigate the relationship between child abuse and oral habits among population-based adolescents in Mongolia. The hypothesis is that child abuse is associated with poor oral habits in adolescents in Mongolia.

## 2. Materials and Methods

### 2.1. Study Design and Setting

This cross-sectional study used data from the “International Comparative Study of Maxillofacial Morphology in Mongoloids”, which is a population-based longitudinal study conducted in 2013–2015 at the Tokyo Medical and Dental University and the Mongolian National University of Medical Sciences. This study has been summarized in previous reports [22,23]. This study was approved by the Ethical Review Board of the Mongolian National University of Medical Science (No. 13-12/1A) and the Tokyo Medical and Dental University (No. D2013-071). This article is structured according to the STROBE (Strengthening the Reporting of Observational Studies in Epidemiology) guidelines for cross-sectional studies. Written informed consent was obtained from the caregivers.

Participants were recruited from public schools in Ulaanbaatar, the capital city of Mongolia. There are 111 public schools and 96 private schools in Ulaanbaatar. Then, two schools, one in an urban area and the other in a suburban area, agreed to participate in the study. From the agreed two schools, two classes were randomly selected for each grade, i.e., from 3rd to 10th grade, aged 7–16 years old.

### 2.2. Dependent Variable: Oral Habits

Parents completed the questionnaire regarding the biting habits and bruxism of their children. Biting habits were assessed using the following questions: “Does your child have a habit of biting your nails/biting your tongue or lips/biting on clothing, pens, pencils, etc.?” The answers to each question were selected from the following options: “never”, “they used to do it before, but they stopped”, and “they still do it.” Each response was categorized into “never” and “used to do it before or still do it.” Moreover, the children were considered to have biting habits if they had at least one of the following three biting habits: biting nails, biting the tongue or lips or biting on clothing.

Bruxism was assessed using the following question: “Does your child grind their teeth?” with the same responses. The answer “they used to do it before, but they stopped” or “they still do” was considered having bruxism.

### 2.3. Independent Variable: Child Abuse

Child abuse was evaluated using a questionnaire completed by the parents. Physical abuse was assessed using the following two questions: “How often do you hit your child on the head?” and “How often do you hit a child in the face?” The responses were provided on a four-point Likert scale with the following possible responses: “often”, “sometimes”, “occasionally”, and “never”. Physical abuse was determined if the respondent selected “often” or “sometimes” for any of the two questions. Psychological abuse was assessed using the following question: “How often do you say something hurtful to your child repeatedly?” Psychological abuse was considered if the responses were “often”, “sometimes”, or “occasionally”. Considering that approximately 50% of the children aged between 1–14 years have experienced psychological or physical discipline in Mongolia, we defined physical and psychological abuse by using the aforementioned criteria [21]. A previous study reported that a history of psychological abuse was associated with low self-esteem, which is a risk factor for nail biting [24]. Based on this study, repeated psychological abuse was considered serious even when occasional [25]. We examined the association between each type of abuse and oral habits and also examined the number of types of child abuse (ranges from 0 to 2) and oral habits.

### 2.4. Covariates

The following covariates were obtained from the questionnaire: the child’s age (continuous), sex (male, female), maternal age (continuous), maternal and paternal education (less than high school, high school, or more), family income level (low, i.e., MNT < 200 thousand; intermediate, i.e., MNT 210 thousand–1800 thousand; high, i.e., MNT ≥ 1801 thousand tugriks; USD 1~MNT 2654), living status with grandparents (yes, no), and order of birth in the family (first, second, or later).

### 2.5. Statistical Analysis

Considering the high prevalence of poor oral habits in adolescents in Mongolia, multivariable Poisson regression models were fitted. Children with missing information on continuous variable were excluded from the analysis. Missing information on categorical variable was coded as a dummy variable and included in the analysis (Table 1). Statistical significance was set at *p* < 0.05. Statistical analyses were performed using the Stata 15 SE software program (Stata Corp LP, College Station, TX, USA).

## 3. Results

A questionnaire survey and oral examination were administered to 892 children and parents (response rate: 86.3%). We excluded individuals who did not submit questionnaires or provide consent (*n* = 34) and those who lacked information regarding oral habits (*n* = 38) or child abuse (*n* = 50). This study included 770 participants in total.

Table 1 summarizes the characteristics of children according to biting habits and bruxism. The rate of prevalence of biting habits and bruxism were 39.0% and 17.5%, respectively. The mean age of the participants was 11.8 (standard deviation = 1.9), and few children were living with their grandparents (22.6%). Biting habits were more common among males (50.7%), while teeth grinding was more common among those living with grandparents (30.4%) and those with the first birth order among their siblings (61.5%).

Table 2 shows the relationship between physical abuse and oral habits. The crude models revealed an association of physical abuse with biting habits (prevalence ratio (PR) = 1.38, 95% confidence interval (CI) = 1.07–1.77), but not bruxism. After adjusting for the age and sex and the socioeconomic status of the parents, physical abuse was significantly associated with biting habits (PR = 1.31, 95% CI = 1.01–1.71), but not bruxism (PR = 1.00, 95% CI = 0.66–1.54).

Table 3 shows the relationship between psychological abuse and oral habits. The crude model revealed an association of psychological abuse with biting habits (PR = 1.44, 95% CI = 1.07–1.95), but not bruxism. After adjusting for the age, sex, and socioeconomic status of the parents, psychological abuse was found to be associated with biting habits, but this association was not significant (PR = 1.34, 95% CI = 0.98–1.83). Bruxism was not found to be associated with psychological abuse (PR = 1.04, 95% CI = 0.68–1.61).

Table 4 summarizes the relationship between the number of types of child abuse with oral habits. In the crude model, biting habits showed a significant positive correlation with the number of types of child abuse. The PR was 1.20 (95% CI = 0.88–1.66) for children who experienced one type of abuse and 1.75 (95% CI = 1.23–2.49) for children who experienced two types of child abuse compared with those without child abuse (*p* for trend: 0.02). After adjusting for the confounding factors, the association remained significant (PR = 1.70, 95% CI = 1.19–2.44) for children with two types of child abuse (*p* for trend: 0.04). Contrastingly, the number of types of child abuse was not associated with bruxism.

## 4. Discussion

The results of this study showed that the association between biting habits and physical abuse was significant for Mongolian adolescents, while it was not significant for psychological abuse; however, the point estimates were similar between physical and psychological abuse (PR = 1.31 and 1.34, respectively), and the confidence interval suggested marginal significance. The dose–response relationship between types of child abuse and biting habits also supports psychological abuse as a potential risk factor for biting habits. In contrast, bruxism was not associated with any of them.

Our findings are consistent with previous reports. A study on children in Iraq aged 10–11 years showed that child abuse was associated with thumb sucking and nail biting, which further suggests that poor oral habits developed to release stress, anxiety, and nervousness [4,6,14]. However, their study did not consider potential confounding factors such as sex, parental education, and family income level. Moreover, they asked the children about their oral habits. In many studies, questionnaires about oral habits have been administered to parents or teachers [4,26,27,28,29].

In the present study, bruxism was not associated with child abuse. This could be attributed to answering the questions by parents. Bruxism occurs mostly during sleep [30] and hence, may not be recognized by them. A systematic review reported variability in prevalence in studies using data answered by parents [30]. A study used questionnaires and polysomnography, which can accurately identify sleep bruxism, to report the prevalence of bruxism. Polysomnography confirmed bruxism in only 47% of the participants with reported bruxism on the questionnaire [31]. Parents who do not abuse their children are more likely to be concerned regarding the health and behavior of their children compared to parents who abuse their children. Therefore, bruxism could have been overreported among children who were not subjected to abuse, which may explain the null association between child abuse and bruxism. Accordingly, since the prevalence varies depending on the measurement, objective measurements, including polysomnography and electromyography, may be required to confirm this association.

This is the first study to examine the association between the number of types of child abuse and oral habits. Children who had suffered multiple victimizations, including various types of abuse, had more trauma symptoms than those who had only suffered the same type of victimization repeatedly [16]. Multiple victimizations have several ill effects on behaviors regarding health, including substance use disorders [32], excessive eating [33], and hypersexuality [34]. The present study found that multiple types of abuse significantly increased the risk of biting habits, which is similar to previous reports.

This study has several limitations. First, child abuse may have been underreported since it was assessed using parental reports. Second, we used a parent-reported questionnaire to assess oral habits; and some parents could have been unaware of the oral habits of their children if they did not spend much time with them. Third, we could not evaluate the association of parenting practices, parent–child relationships, or mental health with oral habits due to a lack of data. Fourth, we did not determine the timing and duration of child abuse, which may influence the results. Fifth, since this was a cross-sectional study, a causal relationship remains unknown. Finally, the generalizability of the finding is limited to rural areas in Mongolia or other countries.

## 5. Conclusions

Children with physical abuse were significantly more likely to have biting habits. Additionally, multiple types of abuse were also found to significantly increase the risk of developing biting habits. Since oral habits are one of the risks of developing oral diseases such as malocclusion and temporomandibular joint disorder, preventing abuse could contribute to the maintenance and improvement of lifelong oral health and the quality of the lives of children.

## Figures and Tables

**Table 1:** Demographic characteristics of the participants according to biting habits.

	ALL	Biting Habits(+)	Biting Habits(−)		Bruxism(+)	Bruxism(−)	
Characteristics	(*n* = 770)	(*n* = 300: 39.0%)	(*n* = 470: 61.0%)	*p*-Value	(*n* = 135: 17.5%)	(*n* = 635: 82.5%)	*p*-Value
	N or Mean (%,SD)	N or Mean (%,SD)	N or Mean (%,SD)		N or Mean (%,SD)	N or Mean (%,SD)	
Sex							
Male	333 (43.3)	152 (50.7)	181 (38.5)	0.001	63 (46.7)	270 (42.5)	0.38
Female	437 (56.7)	148 (49.5)	289 (61.5)		72 (53.3)	365 (57.5)	
Age	11.8 (1.9)	11.7 (1.9)	11.8 (1.9)	0.65	11.8 (2.0)	11.8 (1.9)	0.59
Maternal age	37.6 (5.4)	37.6 (5.8)	37.7 (5.2)	0.70	37.8 (5.2)	37.6 (5.5)	0.73
Family income level							
Low	41 (5.3)	13 (4.3)	28 (6.0)		6 (4.4)	35 (5.5)	
Intermediate	688 (89.4)	272 (90.7)	416 (88.5)	0.37	121 (89.6)	567 (89.3)	0.86
High	27 (3.5)	12 (4.0)	15 (3.2)		6 (4.4)	21 (3.3)	
Missing	14 (1.8)	3 (1.0)	11 (2.3)		2 (1.5)	12 (1.9)	
Maternal Education							
Junior high school	138 (17.9)	56(18.7)	82 (17.5)		23 (17.0)	115 (18.1)	
High school	308 (40.0)	112 (37.3)	196 (41.7)	0.43	47 (34.8)	261 (41.1)	0.16
College or more	315 (40.9)	130 (43.3)	185 (39.4)		65 (48.2)	250 (39.4)	
Missing	9 (1.2)	2 (0.7)	7 (1.5)		0 (0.0)	9 (1.4)	
Paternal Education							
Junior high school	147 (19.1)	60 (20.0)	87 (18.5)		22 (16.3)	125 (19.7)	
High school	319 (41.4)	112 (37.3)	207 (44.0)	0.04	51 (37.8)	268 (42.2)	0.36
College or more	228 (29.6)	104 (34.7)	124 (26.4)		48 (35.6)	180 (28.4)	
Missing	76 (9.9)	24 (8.0)	52 (11.1)		14 (10.4)	62 (9.8)	
Living with grandparents							
Yes	174 (22.6)	78 (26.0)	96 (20.4)		41 (30.4)	133 (20.9)	
No	579 (75.2)	214 (71.3)	365 (77.7)	0.14	89 (65.9)	490 (77.2)	0.02
Missing	17 (2.2)	8 (2.7)	9 (1.9)		5 (3.7)	12 (1.9)	
Number of family	4.6 (1.2)	4.6 (1.2)	4.6 (1.2)	0.86	4.6 (1.4)	4.6 (1.2)	0.66
Birth order							
First	402 (52.2)	169 (56.3)	233 (49.6)		83 (61.5)	319 (50.2)	
Second or later	352 (45.7)	127 (42.3)	225 (47.9)	0.13	49 (36.3)	303 (47.7)	0.05
Missing	16 (2.1)	4 (1.3)	12 (2.6)		3 (2.2)	13 (2.1)	
Physical abuse							
Yes	173 (22.5)	85 (28.3)	88 (18.7)	0.002	30 (22.2)	143 (22.5)	0.94
No	597 (77.5)	215 (71.7)	382 (81.3)		105 (77.8)	492 (77.5)	
Psychological abuse							
Yes	590 (76.6)	248 (82.7)	342 (72.8)	0.002	107 (79.3)	483 (76.1)	0.43
No	180 (23.4)	52 (17.3)	128 (27.2)		28 (20.7)	152 (23.9)	

Abbreviations: SD, standard deviation.

**Table 2:** Associations between physical abuse and oral habits (N = 761 ^a^).

	Biting Habits		Bruxism	
	Crude	Adjusted	Crude	Adjusted
	PR (95% CI)	PR (95% CI)	PR (95% CI)	PR (95% CI)
Physical abuse	1.38 (1.07–1.77) *	1.31 (1.01–1.71) *	0.99 (0.66–1.48)	1.00 (0.66–1.54)
Psychological abuse				
No	Ref	Ref	Ref	Ref
Yes	1.44 (1.07–1.95)	1.34 (0.98–1.83)	1.17 (0.77–1.77)	1.04 (0.68–1.61)
Age	0.99 (0.93–1.05)	1.00 (0.94–1.06)	1.02 (0.94–1.12)	1.02 (0.92–1.12)
Maternal age	1.00 (0.98–1.02)	1.01 (0.98–1.03)	1.00 (0.97–1.04)	1.03 (0.99–1.07)
Sex				
Male	Ref	Ref	Ref	Ref
Female	0.75 (0.59–0.94)	0.76 (0.60–0.95)	0.88 (0.62–1.23)	0.90 (0.64–1.27)
Maternal Education				
Junior high school	Ref	Ref	Ref	Ref
High school	0.90 (0.65–1.24)	0.92 (0.64–1.33)	0.92 (0.56–1.51)	0.84 (0.48–1.47)
College or more	1.02 (0.74–1.39)	0.91 (0.60–1.38)	1.24 (0.77–1.99)	1.01 (0.54–1.89)
Paternal Education				
Junior high school	Ref	Ref	Ref	Ref
High school	0.87 (0.64–1.20)	0.95 (0.66–1.35)	1.07 (0.65–1.76)	1.15 (0.66–2.02)
College or more	1.12 (0.81–1.54)	1.17 (0.78–1.77)	1.39 (0.84–2.30)	1.29 (0.68–2.44)
Missing	0.77 (0.48–1.25)	0.76 (0.46–1.28)	1.26 (0.65–2.47)	1.17 (0.57–2.42)
Family income level				
Low	Ref	Ref	Ref	Ref
Intermediate	1.26 (0.71–2.24)	1.18 (0.64–2.19)	1.12 (0.49–2.54)	1.08 (0.45–2.60)
High	1.41 (0.63–3.13)	1.35 (0.58–3.15)	1.41 (0.45–4.36)	1.34 (0.40–4.47)
Missing	0.53 (0.12–2.36)	0.50 (0.11–2.28)	1.06 (0.21–5.23)	1.23 (0.24–6.25)
Living with grandparents				
No	Ref	Ref	Ref	Ref
Yes	1.22 (0.94–1.59)	1.23 (0.94–1.60)	1.53 (1.06–2.22)	1.49 (1.02–2.19)
Missing	1.27 (0.63–2.57)	1.19 (0.58–2.44)	1.89 (0.77–4.65)	2.07 (0.82–5.22)
Birth order				
First	Ref	Ref	Ref	Ref
Second or later	0.86 (0.68–1.09)	0.92 (0.70–1.22)	0.67 (0.47–0.96)	0.63 (0.42–0.97)
Missing	0.51 (0.16–1.59)	0.53 (0.17–1.68)	1.03 (0.32–3.25)	1.02 (0.32–3.32)

Abbreviations: PR, prevalence ratios. ^a^ Children with lack of information on maternal education were excluded to prevent perfect prediction. * *p* < 0.05.

**Table 3:** Associations between psychological abuse and oral habits (N = 761 ^a^).

	Biting Habits		Bruxism	
	Crude	Adjusted	Crude	Adjusted
	PR (95% CI)	PR (95% CI)	PR (95% CI)	PR (95% CI)
Psychological abuse	1.44 (1.07–1.95) *	1.34 (0.98–1.83)	1.16 (0.77–1.77)	1.04 (0.68–1.61)
Physical abuse				
No	Ref	Ref	Ref	Ref
Yes	1.38 (1.07–1.77)	1.31 (1.01–1.71)	0.99 (0.66–1.48)	1.00 (0.66–1.54)
Age	0.99 (0.93–1.05)	1.00 (0.94–1.06)	1.02 (0.94–1.12)	1.02 (0.92–1.112)
Maternal age	1.00 (0.98–1.02)	1.01 (0.98–1.03)	1.00 (0.97–1.04)	1.03 (0.99–1.07)
Sex				
Male	Ref	Ref	Ref	Ref
Female	0.75 (0.59–0.94)	0.76 (0.60–0.95)	0.88 (0.62–1.23)	0.90 (0.64–1.27)
Maternal Education				
Junior high school	Ref	Ref	Ref	Ref
High school	0.90 (0.65–1.24)	0.92 (0.64–1.33)	0.92 (0.56–1.51)	0.84 (0.48–1.47)
College or more	1.02 (0.74–1.39)	0.91 (0.60–1.38)	1.24 (0.77–1.99)	1.01 (0.54–1.89)
Paternal Education				
Junior high school	Ref	Ref	Ref	Ref
High school	0.87 (0.64–1.20)	0.95 (0.66–1.35)	1.07 (0.65–1.76)	1.15 (0.66–2.02)
College or more	1.12 (0.81–1.54)	1.17 (0.78–1.77)	1.39 (0.84–2.30)	1.29 (0.685–2.44)
Missing	0.77 (0.48–1.25)	0.76 (0.46–1.28)	1.26 (0.65–2.47)	1.17 (0.57–2.42)
Family income level				
Low	Ref	Ref	Ref	Ref
Intermediate	1.26 (0.71–2.24)	1.18 (0.64–2.19)	1.12 (0.49–2.54)	1.08 (0.459–2.60)
High	1.41 (0.63–3.13)	1.35 (0.58–3.15)	1.41 (0.45–4.36)	1.34 (0.40–4.47)
Missing	0.53 (0.12–2.36)	0.50 (0.11–2.28)	1.06 (0.21–5.23)	1.23 (0.24–6.25)
Living with grandparents				
No	Ref	Ref	Ref	Ref
Yes	1.22 (0.94–1.59)	1.23 (0.94–1.60)	1.53 (1.06–2.22)	1.49 (1.02–2.19)
Missing	1.27 (0.63–2.57)	1.19 (0.58–2.44)	1.89 (0.77–4.65)	2.07 (0.82–5.22)
Birth order				
First	Ref	Ref	Ref	Ref
Second or later	0.86 (0.68–1.09)	0.92 (0.70–1.22)	0.67 (0.47–0.96)	0.63 (042–0.97)
Missing	0.51 (0.16–1.59)	0.53 (0.17–1.68)	1.03 (0.32–3.25)	1.02 (0.32–3.32)

Abbreviations: PR, prevalence ratios. ^a^ Children with lack of information on maternal education were excluded to prevent perfect prediction. * *p* < 0.05.

**Table 4:** Associations between the number of types of child abuse history and oral habits (N = 761 ^a^).

	Biting Habits		Bruxism	
	Crude	Adjusted	Crude	Adjusted
	PR (95% CI)	PR (95% CI)	PR (95% CI)	PR (95% CI)
No. of child abuse history				
0	Ref	Ref	Ref	Ref
1	1.20 (0.88–1.66)	1.16 (0.84–1.61)	1.10 (0.71–1.71)	0.98 (0.62–1.54)
2	1.75 (1.23–2.49) *	1.70 (1.19–2.44) *	1.13 (0.66–1.92)	1.05 (0.61–1.80)
Age		1.00 (0.94–1.07)		1.02 (0.92–1.12)
Maternal age		1.01 (0.98–1.03)		1.03 (0.99–1.07)
Sex				
Male		Ref		Ref
Female		0.76 (0.60–0.96)		0.91 (0.64–1.27)
Maternal Education				
Junior high school		Ref		Ref
High school		0.93 (0.64–1.34)		0.84 (0.48–1.48)
College or more		0.94 (0.62–1.41)		1.02 (0.55–1.91)
Paternal Education				
Junior high school		Ref		Ref
High school		0.96 (0.67–1.37)		1.16 (0.66–2.03)
College or more		1.19 (0.79–1.79)		1.29 (0.68–2.45)
Missing		0.77 (0.46–1.29)		1.18 (0.57–2.43)
Family income level				
Low		Ref		Ref
Intermediate		1.20 (0.65–2.22)		1.09 (0.45–2.62)
High		1.39 (0.59–3.23)		1.36 (0.41–4.54)
Missing		0.52 (0.17–1.69)		1.25 (0.25–6.36)
Living with grandparents				
No		Ref		Ref
Yes		1.23 (0.94–1.60)		1.49 (1.02–2.20)
Missing		1.24 (0.60–2.53)		2.11 (0.84–5.33)
Birth order				
First		Ref		Ref
Second or later		0.93 (0.70–1.22)		0.63 (0.42–0.97)
Missing		0.53 (0.17–1.69)		1.03 (0.32–3.32)

Abbreviations: PR, prevalence ratios. ^a^ Children with lack of information on maternal education were excluded to prevent perfect prediction. * *p* < 0.05.

## Data Availability

The data that support the findings of this study are available on request from the corresponding author. The data are not publicly available due to privacy or ethical restrictions.
